# Expression levels of filaggrin-2 in relation to drip loss in pigs

**DOI:** 10.5713/ab.21.0220

**Published:** 2021-10-21

**Authors:** Autchara Kayan, Nunyarat Koomkrong

**Affiliations:** 1 Department of Animal Science, Faculty of Agriculture, Kasetsart University, Bangkok 10900, Thailand; 2 Department of Animal Science, Faculty of Science and Technology, Suratthani Rajabhat University, Suratthani 84100, Thailand

**Keywords:** Drip Loss, Expression, Filaggrin-2 (FLG2), Pig

## Abstract

**Objective:**

The aim of this study was to investigate the expression level of filaggrin-2 (FLG2) in correlation with drip loss.

**Methods:**

The muscle samples were randomly taken from a local meat supplier. Samples were taken from *Longissimus lumborum* muscles to evaluate the drip loss (n = 100). Five muscles per group (low and high drip loss) were selected to evaluate FLG2 mRNA and protein expression levels.

**Results:**

mRNA of *FLG2* gene was not significantly different in pigs with different levels of drip loss (p>0.05). Statistical analysis revealed that FLG2 protein expression levels were significantly different between the drip loss groups. Western blot revealed that the high drip loss group had higher FLG2 protein expression level than the low drip loss group (p<0.001). Moreover, immunohistochemistry revealed the high signal intensity was on the muscle cell membrane and cytoplasm.

**Conclusion:**

FLG2 protein might play roles in drip loss of pork and will provide the basis for information to improving meat quality traits in pigs.

## INTRODUCTION

Drip loss can be used to determine water-holding capacity in raw meat and it has high importance in pig meat production due to its financial implications including the loss of weight, reducing acceptance and causing rejection by consumers [1]. The drip loss for pork was found to range from 2.2% to 12.6% depending on meat pH, carcass temperature, postmortem metabolism and muscle fiber structural changes [2]. Some of the proteins in muscle tissue related to drip loss [3,4] have been implicated by their expression in proteomic studies. Proteomic study makes an important contribution towards a more detailed molecular view of the processes behind water-holding capacity [5]. Some of the proteins have been associated with meat quality traits [4,6]. Filaggrin-2 (FLG2) is a member of the S100-fused type protein family and a key protein implicated in epidermal barrier functions [7]. The biological process of FLG2 is involved in structural constituent of cytoskeleton [5]. Cytoskeleton functions to form a framework within the muscle fiber, connecting the myofibrils to the sarcolemma and keeping the complex arrangement of the contractile elements [8]. The state of the cytoskeleton can influence the loss of muscle exudates known as drip loss, which originate from shrinkage of myofibrils after death [9,10]. The fibers shrink as their constituent myofibrils shrink and the water that is left behind accumulates first around the perimysial network and later around the endomysial network, giving rise to two extracellular water compartments. In pale soft exudative (PSE) meat the myofibrils shrink about twice as much as in normal meat [11]. Moreover, FLG2 protein is a substrate of calpain-1 [7,12], which plays a major role in regulating proteolysis of cytoskeletal proteins under postmortem conditions [10]. It has been found that increased degradation of the intermediate filament protein may compensate for some of the shrinkage of muscle cells [13]. In pigs, FLG2 was lower in abundance in low drip loss compared to intermediate drip loss [5] and high drip loss [14]. In human, FLG2 protein is variably expressed in the stratum corneum of dermatitic skin. Its expression is minimized in those with acute spongiotic dermatitis and in association with a brisk inflammatory infiltrate [15]. Moreover, FLG2 is essential for normal cell-cell adhesion in the cornified cell layers. FLG2 was found to be expressed throughout the cornified cell layers and to colocalize with corneodesmosin that plays a crucial role in maintaining cell-cell adhesion in this region of the epidermis [16]. This study combines information on protein localization, mRNA, and protein abundance. The study of proteins expressed at the muscle fiber related to drip loss. The quantitative study of the expression of FLG2 proteins and their localization is also vital for identifying the protein role in adequate amount, in the correct localization and interacting between muscle function on drip loss. In our study, we have investigated the expression of FLG2 in *Longissimus lumborum* muscle, to explore the possible presence of alterations that could be useful in studying the biology of *FLG2* gene on drip loss.

## MATERIALS AND METHODS

### Animals and muscle sampling

A total of 100 muscle samples of three crossbred pigs (Duroc× [Large White×Landrace]) were randomly collected from a local meat supplier in Thailand. All pigs were slaughtered at about 6 months of age and the average body weight was 112.13±4.81 kg according to standard slaughtering procedures of DLD (Department of Livestock Development, Thailand). After electrical stunning, carcasses were scalded, cleaned, eviscerated, and split. The muscle samples were immediately taken from the *Longissimus lumborum* between 13th/14th rib to evaluate drip loss and the muscle samples were kept at −20°C for protein expression and −80°C for mRNA expression until subsequent analysis.

### Drip loss analysis

Drip loss was scored based on a bag method with a size-standardized sample from *Longissimus lumborum* muscle at the 13th to 14th ribs collected at 24 h post-mortem. The samples were cut to a 2.5 cm thick slice of muscle, weighed, suspended in a plastic bag, held at 4°C for 48 h, and thereafter re-weighed. Drip loss was calculated as a percentage loss of weight [17]. The divergent drip losses were categorized based on the previous studies [4,5,18–20]. In this study muscle samples included low drip loss (≤1.47%) and high drip loss (≥6.25%) groups.

### mRNA expression of FLG2

Total RNA was isolated from 20 mg of the *Longissimus lumborum* muscle of low and high drip loss groups (n = 5 per group) by using QIAamp RNA Mini Kit (Qiagen, Courtaboeuf, France) according to manufacturer recommendations. The purity of the extracted RNA was measured using the NanoDrop spectrophotometer. Real-time polymerase chain reaction (PCR) analysis was run using MyGo Pro real-time PCR instrument (IT-IS Life Science Ltd, Middlesbrough, UK) with reaction mixture using QuantiNova SYBR Green RT-PCR Kit (Qiagen, Hilden, Germany), consisting of 10 μL of 2× QuantiNova SYBR Green RT-PCR Master Mix, 1 μL of each 10 μM (0.5 μM) forward and reverse primer, 0.2 μL of QN SYBR Green RT Mix, 5 μL of template and 2.8 μL of nuclease-free water was made to a total volume of 20 μL. A two-step amplification program was pre-denaturation at 95°C for 2 min, followed by 40 cycles of denaturation at 95°C for 5 s and annealing and extension at 60°C for 10 s. As a technical replication, all samples were repeated and the mean of the two replications was finally used. Results were reported as the relative expression level compared after normalization of the transcript level using the housekeeping gene TATA sequence binding protein (*TBP*). PCR Primers were designed using the Primer3 software [21] and are shown in Table 1.

### Protein expression of FLG2 protein by western blot

The samples were selected from 20 mg of the *Longissimus lumborum* muscle of low and high drip loss groups (n = 5 per group). Extraction of total muscle proteins was by TRI Reagent (Sigma-Aldrich, St. Louis, MO, USA) according to the manufacturer’s instructions. Muscle protein concentration was determined by spectrophotometry. The muscle protein samples (30 μg total) were loaded on each well of a sodium dodecyl sulfate – 10% polyacrylamide gel at 100 V for 190 min in the Mini-PROTEIN II cell (Bio-Rad Laboratories, Hercules, CA, USA). Protein was then transferred to a polyvinylidene fluoride (PVDF) membrane (Bio-Rad Laboratories, USA) with Mini Trans-Blot^®^ cell (Bio-Rad Laboratories, Beijing, China) in transfer buffer (25 mM Tris, 1.4% glycine and 20% methanol) at 150 mA for 90 min. The PVDF membrane was incubated for 1 h at room temperature with anti-FLG2 antibody (diluted 1:800; LS-C293945; LifeSpan BioSciences, Inc., Seattle, WA, USA) in blocking buffer (20 mM Tris pH 7.5, 150 mM NaCl, 0.05% Tween-20 and 1% polyvinylpyrrolidone). Non-specific binding of antibody was washed off with six changes of 0.05% phosphate buffered saline with Tween-20, followed by detection with 1:10,000 diluted horseradish peroxidase (HRP) conjugated secondary Goat anti-Rabbit IgG-HRP (sc-2004; Santa Cruz Biotechnology, Inc., Santa Cruz, CA, USA) at room temperature for 1 h using the Clarity Western ECL Substrate Detechtion System (Bio-Rad Laboratories, Hercules, CA, USA) and visualized by using Omega Lum G imager (Aplegen Gel Company, San Francisco, CA, USA). Glyceraldehyde-3-phosphate dehydrogenase (GAPDH) antibody (I-19) (diluted 1:1,000; sc-48166; Santa Cruz Biotechnology, Inc., USA) was used as a loading control and for normalization. Relative band intensities were compared by determining the ratio of the area densities of FLG2 to GAPDH bands for each lane using Image-J software (National Institute of Mental Health, Bethesda, MD, USA).

### Immunohistochemical detection of FLG2 protein

The muscle samples (*Longissimus lumborum*) of low and high drip loss groups were cut into 0.5×0.5×1.0 cm pieces within 45 min post-mortem after carcass bleeding, then immediately fixed in 10% buffered neutral formalin solution for 24 hours. After fixation of the specimen, dehydrated in alcohol, cleared in xylene, infiltrated and finally embedded in paraffin [22]. The paraffin sections were cut at 3 μm thickness and mounted on positively charged slides. Then, the sections were deparaffinized and antigens were retrieved by incubating the slides in citrate buffer at a pH of 6.0 at 95°C. Endogenous peroxidase activity was quenched with 3% H_2_O_2_ in distilled water, followed by blocking of the nonspecific background using 2% bovine serum albumin at room temperature (RT). Subsequently, the slides were incubated separately overnight at 4°C in humidified chamber with rabbitanti-FLG2 antibody (diluted 1:50; 140329; US Biological Life Sciences, Swampscott, MA, USA). The slides were then incubated with a HRP conjugated goat anti-rabbit antibody (EnVision, Dako, Denmark) at RT for 1 h, followed by development with diaminobenzidine chromogen (Invitrogen, Carlsbad, CA, USA). The sections were counterstained with hematoxylin. PBS (0.01 M) was used as a negative control. Stained cross-sections were viewed and photographed with a light microscope (Olympus FSX100; Olympus, Tokyo, Japan) at 10× objective lens and a 10× eye piece.

### Statistical analysis

Statistical analysis of the differences between drip loss groups was evaluated by t-tests of SAS (SAS Inst. Inc., Cary, NC, USA). Values of p<0.05 were considered to indicate statistically significant differences. The results are presented as least squares means with the standard errors.

## RESULTS

### Drip loss

The drip loss variation from all samples was between 0.00% to 9.84%. The mean value, standard deviations, minimum and maximum of drip loss was 3.61, 1.86, 0.00, and 9.84, respectively [23]. The mean value of low and high drip loss groups was 1.14%±0.64% and 7.72%±1.48%, respectively. The drip loss was negatively correlated with pH 24 h post-mortem (r = −0.92, p<0.001) (data not shown).

### mRNA expression of FLG2

**Figure 1:**
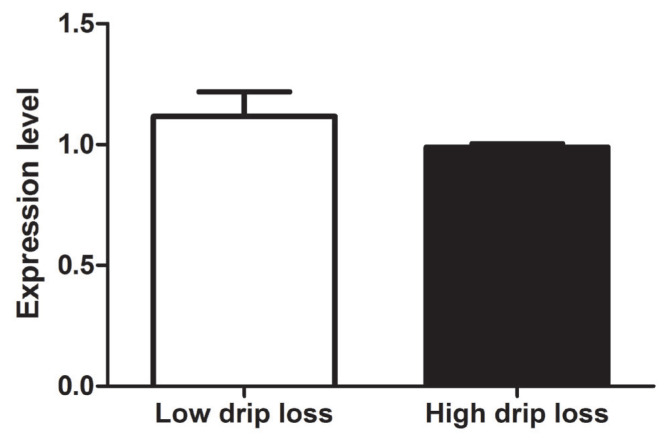
Expression level of *FLG2* gene transcript in *Longissimus lumborum* muscle between low and high drip loss groups: RT-qPCR analysis of *FLG2* gene expression in the *Longissimus lumborum* muscle from low drip loss (≤1.47%) and high drip loss (≥6.25%) groups (n = 5 per group) in pigs. TBP was used as a reference for normalization. The bars represent the means±standard error of the means. The mRNA expression of *FLG2* gene was no significant difference between groups (p>0.05). *FLG2*, filaggrin-2; RT-qPCR, real-time quantitative polymerase chain reaction; TBP, TATA sequence binding protein.

Quantitative real-time PCR analysis showed the abundance of *FLG2* transcript with divergent drip loss in pig muscle. The results showed that the *FLG2* mRNA expression was not significantly different between pigs with high drip loss muscle and low drip loss muscle (p>0.05) (Figure 1). The abundance of *FLG2* transcript of low and high drip loss groups was 1.117±0.102 and 0.989±0.014, respectively.

### Protein expression of FLG2

**Figure 2:**
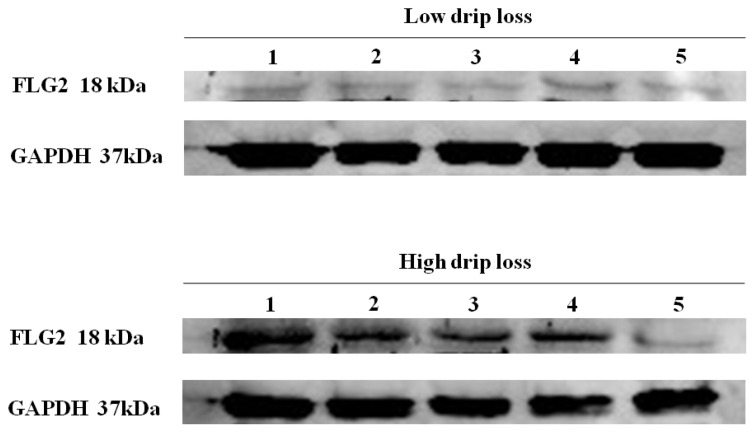
FLG2 protein expression in low and high drip loss groups: Western blot analysis of FLG2 and GAPDH protein expression in the *Longissimus lumborum* muscle from low drip loss (≤1.47%) and high drip loss (≥6.25%) groups (n = 5 per group) in pigs. GAPDH was used as a reference for normalization. Relative band intensities were compared by determining the ratio of the area densities of FLG2 to GAPDH bands for each lane. The high drip loss group had higher FLG2 protein expression levels than the low drip loss group. FLG2, filaggrin-2; GAPDH, glyceraldehyde-3-phosphate dehydrogenase.

In terms of protein expressions, there were significant differences in FLG2 protein expression levels between the drip loss groups. The high drip loss group had higher FLG2 protein expression levels than the low drip loss group (Figure 2). The differences in optical density of the FLG2 protein bands between the drip loss groups were significantly different. The high drip loss group had higher optical density values than the low drip loss group. The optical density values of low and high drip loss groups were 0.260±0.021 and 0.758±0.057, respectively (p<0.001).

### FLG2 protein localization

**Figure 3:**
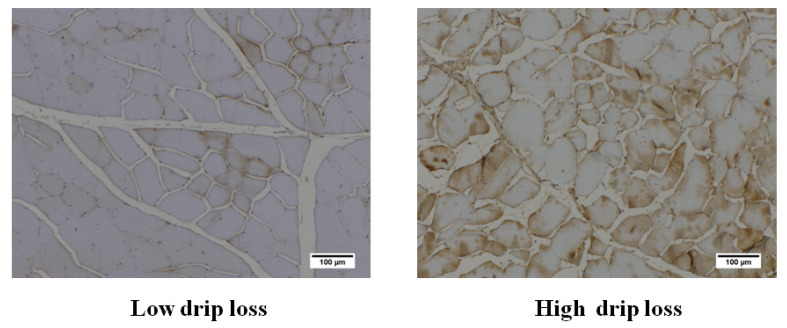
FLG2 protein expression in muscle fiber of low and high drip loss groups: The immunohistochemical detection of FLG2 protein in formalin-fixed paraffin-embedded samples from the *Longissimus lumborum* muscle from low drip loss (≤1.47%) and high drip loss (≥6.25%) groups in pigs. Stained cross-sections were imaged with a light microscope at 100×. The higher signal intensity was observed on the muscle cell membrane and periphery of the cytoplasm in high drip loss group. FLG2, filaggrin-2.

FLG2 protein was detected by immunohistochemistry in the muscle fiber samples. The higher signal intensity was observed on the muscle cell membrane and periphery of the cytoplasm in high drip loss group. The staining was more pronounced in the muscle fiber of high drip loss group (Figure 3).

## DISCUSSION

The water-holding capacity is affected by many factors in the whole meat production chain [9]. Drip loss has a wide range between 0% to 15% in pork [24]. The range of drip loss in this study was lower than other studies, which was reported between 0.21% to 16.51% [25], 1.28% to 16.08% [26] and 2.20% to 20.7% [27]. The pH at 24 h postmortem in this study is high. The negative correlation between pH and drip loss has been reported [28,29]. The drip loss varies according to postmortem metabolism as a result of ATP degradation and the rate of acidification [30]. A faster pH decline causes denaturation of sarcoplasmic and myofibrillar proteins, resulting in reduced water holding capacity [31]. In this study, the highest proportion of samples (56% of all samples) was lower than the 3% drip loss that was defined in normal meat [30,32]. The pork with a drip loss higher than 4% (44% of all samples) was likely to be an incidence of PSE meat [30]. The drip loss variations are affected by many factors in the whole meat production chain including physiological factors, management conditions and processing factors [9]. The genetic correlation between WHC and drip loss traits is high. There is also a correlation to other meat quality traits, such as pH value, cooking loss, reflectance, etc. [33]. The heritability was 0.28± 0.09 [34]. In general terms, each meat trait is under single or multiple gene control. However, expression of genotype depends to a great extent on environmental conditions, which can differ. These conditions, from the aspect of meat quality, include various pre-slaughter conditions and post-slaughter factors. Interactions between genes and environment occur to a greater or lesser degree, and it is difficult to separate each of them [35]. Consequently, expression of these traits in this study might be influenced from the interaction between the traits and environment condition [36].

Proteins are the major product of life process and might reflect gene function more directly than mRNA. In addition, some messages are transcribed but not translated, thus the number of mRNA copies does not necessarily reflect the number of functional protein molecules [37]. Early postmortem changes of muscle proteins are key factors influencing the loss of water in meat and proteolytic degradation results in shrinking of muscle cells and drip loss [38]. However, FLG2 is a key protein implicated in the epidermal barrier functions. Both display a related structural organization, an identical pattern of expression and localization in human epidermis, and proteolytic processing of a large precursor [7]. The biological process of FLG is involved in structural constituents of the cytoskeleton [5], that relates to water-holding capacity of meat. Previous studies found that FLG2 protein was lower in abundance in low drip loss compared to intermediate drip loss [5] and high drip loss [14] in pork muscle exudate. The same result as this study, the expression level of FLG2 protein was correlated to drip loss in these commercial crossbred pigs. A high expression level and the mean values of optical density of FLG2 protein were detected in the high drip loss group. The higher signal intensity was observed on the sarcolemma and periphery of the sarcoplasm in high drip loss group by immunohistochemistry due to the basic unit of contraction being the sarcomere, which is comprised of a plethora of structural and regulatory proteins. The sarcomere is tethered to the sarcolemma, the membrane surrounding the myofibril by another cytoskeletal assembly [39]. The same result as the previous reports, FLG2 protein was lower in abundance in low drip loss compared to intermediate drip loss [5] and high drip loss [14] in pork muscle exudate. This might be due to FLG2 protein being a substrate of calpain 1 [7,12] which plays a major role in regulating proteolysis of cytoskeletal proteins under postmortem conditions [10]. Because the activation rate of calpain 1 is associated with proteolysis of cytoskeletal proteins and therefore could play a role in drip loss [18]. Duroc×Landrace× Yorkshire crossbred pork had high calpain 1 mRNA expression, higher calpain 1 activity and increased rate of muscle protein proteolysis, resulting in the lower pH values and higher drip loss than commercial Meishan pork [40]. The functions of the cytoskeleton include forming a framework within the muscle fiber, connecting the myofibrils to the sarcolemma and keeping the complex arrangement of the contractile elements [8]. Thus, the state of the cytoskeleton can influence the loss of exudates [10] due to drip loss originating from shrinkage of fiber after death [9]. The fibers shrink as their constituent myofibrils shrink and the water that is left behind accumulates first around the perimysial network and later around the endomysial network, giving rise to two extracellular water compartments. In PSE meat, the myofibrils shrinkage was found about twice as much as in normal meat [11]. The degradation of the cytoskeleton slowly removes the linkage between lateral shrinkage of myofibrils and shrinkage of entire muscle fibers, so removing the force that causes flow into the extracellular space [41]. Moreover, it has been found that increased degradation of the intermediate filament protein may compensate for some of the shrinkage of the muscle cell due to the drop in pH [13]. Water loss from the muscle is impacted by a variety of the structural changes of muscle [42]. Therefore, the present results indicated high levels of FLG2 was associated with cell function and increasing of water loss, and it could act as indicator for poor water holding capacity of meat.

## CONCLUSION

This study revealed that the expression level of FLG2 protein and immunohistochemistry was correlated to drip loss in these commercial crossbred pigs. The high expression level of FLG2 protein was detected in the high drip loss group. Therefore, these results might be used to improve water-holding capacity in terms of drip loss in pork.

## Figures and Tables

**Table 1:** Real-time quantitative polymerase chain primer sequences

Name		Sequence	Amplicon length (bp)	Annealing temperature (°C)
*FLG2*	Forward	TCCAGTGACAGTGAAAGGCA	234	60
	Reverse	CCTGTGACTGCGTGTGAAAA		
*TBP*	Forward	GATGGACGTTCGGTTTAGG	124	60
	Reverse	AGCAGCACAGTACGAGCAA		

*FLG2*, filaggrin-2; *TBP*, TATA sequence binding protein.

## References

[1] Ocampo Ibáñez ID, Bermúdez F, Díaz H (2009). Effect of storage time, muscle type, and animal genotype on drip loss in raw pork. Acta Agron.

[2] Schäfer A, Rosenvold K, Purslow PP, Andersen HJ, Henckel P (2002). Physiological and structural events post mortem of importance for drip loss in pork. Meat Sci.

[3] Pas MFt, Kruijt L, Pierzchala M (2013). Identification of proteomic biomarkers in M. Longissimus dorsi as potential predictors of pork quality. Meat Sci.

[4] Zhang M, Wang D, Geng Z (2014). The level of heat shock protein 90 in pig Longissimus dorsi muscle and its relationship with meat pH and quality. Food Chem.

[5] Luca AD, Elia G, Hamill R, Mullen AM (2013). 2D DIGE proteomic analysis of early post mortem muscle exudate highlights the importance of the stress response for improved water-holding capacity of fresh pork meat. Proteomics.

[6] Luca AD, Elia G, Mullen AM, Hamill RM (2013). Monitoring post mortem changes in porcine muscle through 2-D DIGE proteome analysis of Longissimus muscle exudate. Proteome Sci.

[7] Hsu C-Y, Henry J, Raymond A-A (2011). Deimination of human filaggrin-2 promotes its proteolysis by calpain 1. J Biol Chem.

[8] Warriss PD (2010). Meat science: an introductory text.

[9] Hertog-Meischke MJAd, Laack RJLMv, Smulders FJM (1997). The water-holding capacity of fresh meat. Vet Q.

[10] Huff-Lonergan E, Lonergan SM (2005). Mechanisms of water-holding capacity of meat: The role of postmortem biochemical and structural changes. Meat Sci.

[11] Offer G, Knight P, Jeacocke R (1989). The structural basis of the water-holding, appearance and toughness of meat and meat products. Food Microstruct.

[12] Pendaries V, Lamer ML, Cau L (2015). In a three-dimensional reconstructed human epidermis filaggrin-2 is essential for proper cornification. Cell Death Dis.

[13] Melody JL, Lonergan SM, Rowe LJ, Huiatt TW, Mayes MS, Huff-Lonergan E (2004). Early postmortem biochemical factors influence tenderness and water-holding capacity of three porcine muscles. J Anim Sci.

[14] Luca AD, Hamill RM, Mullen AM, Slavov N, Elia G (2016). Comparative proteomic profiling of divergent phenotypes for water holding capacity across the post mortem ageing period in porcine muscle exudate. PLoS ONE.

[15] Seykora J, Dentchev T, Margolis DJ (2015). Filaggrin-2 barrier protein inversely varies with skin inflammation. Exp Dermatol.

[16] Mohamad J, Sarig O, Godsel LM (2018). Filaggrin 2 deficiency results in abnormal cell-cell adhesion in the cornified cell layers and causes peeling skin syndrome type A. J Invest Dermatol.

[17] Honikel KO, Kim CJ, Hamm R, Roncales P (1986). Sarcomere shortening of prerigor muscles and its influence on drip loss. Meat Sci.

[18] Bee G, Anderson AL, Lonergan SM, Huff-Lonergan E (2007). Rate and extent of pH decline affect proteolysis of cytoskeletal proteins and water-holding capacity in pork. Meat Sci.

[19] Kayan A, Uddin MJ, Cinar MU (2011). Investigation on interferon alpha-inducible protein 6 (IFI6) gene as a candidate for meat and carcass quality in pig. Meat Sci.

[20] Traore S, Aubry L, Gatellier P (2012). Higher drip loss is associated with protein oxidation. Meat Sci.

[21] Rozen S, Skaletsky H, Misener S, Krawetz SA (2000). Primer3 on the WWW for general users and for biologist programmers. Bioinformatics methods and protocols. Methods in Molecular Biology, vol 132.

[22] Khoshoii AA, Mobini B, Rahimi E (2013). Comparison of chicken strains: Muscle fibre diameter and numbers in Pectoralis superficialis muscle. Glob Vet.

[23] Koomkrong N, Gongruttananun N, Boonkaewwan C, Noosud J, Theerawatanasirikul S, Kayan A (2017). Fiber characteristics of pork muscle exhibiting different levels of drip loss. Anim Sci J.

[24] Adzitey F, Nurul H (2011). Pale soft exudative (PSE) and dark firm dry (DFD) meats: causes and measures to reduce these incidences - a mini review. Int Food Res J.

[25] Borchers N, Otto G, Kalm E (2007). Genetic relationship of drip loss to further meat quality traits in purebred Piétrains. Arch Tierz.

[26] Mörlein D, Link G, Werner C, Wicke M (2007). Suitability of three commercially produced pig breeds in Germany for a meat quality program with emphasis on drip loss and eating quality. Meat Sci.

[27] Warriss PD, Brown SN (1987). The relationships between initial pH, reflectance and exudation in pig muscle. Meat Sci.

[28] Ryu YC, Kim BC (2005). The relationship between muscle fiber characteristics, postmortem metabolic rate, and meat quality of pig longissimus dorsi muscle. Meat Sci.

[29] Sieczkowska H, Andrzejczuk A, Zybert A (2017). Usefulness of pork meat quality classes criteria in assessing of its culinary value. Sci Ann Polish Soc Anim Prod.

[30] Żelechowska E, Przybylski W, Jaworska D, Santé-Lhoutellier V (2012). Technological and sensory pork quality in relation to muscle and drip loss protein profiles. Eur Food Res Technol.

[31] Scheffler TL, Gerrard DE (2007). Mechanisms controlling pork quality development: The biochemistry controlling postmortem energy metabolism. Meat Sci.

[32] Jůzl M, Šulcerová H, Gregor T (2012). The relationship between colour and other meat quality traits in Czech Large White pigs. Maso Int - J Food Sci Technol.

[33] Jennen D, Brings A, Liu G (2007). Genetic aspects concerning drip loss and water-holding capacity of porcine meat. J Anim Breed Genet.

[34] Willson HE, Rojas de Oliveira H, Schinckel AP, Grossi D, Brito LF (2020). Estimation of genetic parameters for pork quality, novel carcass, primal-cut and growth traits in Duroc pigs. Animals.

[35] Warner R, Greenwood P, Pethick D, Ferguson DM (2010). Genetic and environmental effects on meat quality. Meat Sci.

[36] Zaman R, Nassir HM, Abdurrazq NB, Salleh HM, Rahman MT (2012). Effects of different methods of slaughtering on protein expression in chicken meat. IIUM Eng J.

[37] Guo Y, Xiao P, Lei S (2008). How is mRNA expression predictive for protein expression? A correlation study on human circulating monocytes. Acta Biochim Biophys Sin.

[38] Bernevic B, Petrea BA, Galetskiya D (2011). Degradation and oxidation postmortem of myofibrillar proteins in porcine skeleton muscle revealed by high resolution mass spectrometric proteome analysis. Int J Mass Spectrom.

[39] Henderson CA, Gomez CG, Novak SM, Mi-Mi L, Gregorio CC (2017). Overview of the muscle cytoskeleton. Compr Physiol.

[40] Wang J, Yan X-L, Liu R, Fu Q, Zhou G, Zhang W (2016). Differences in calpain system, desmin degradation and water holding capacity between commercial Meishan and Duroc × Landrace × Yorkshire crossbred pork. Anim Sci J.

[41] Kristensen L, Purslow PP (2001). The effect of ageing on the water-holding capacity of pork: role of cytoskeletal proteins. Meat Sci.

[42] Zhang M, Wang D, Geng Z (2017). Differential expression of heat shock protein 90, 70, 60 in chicken muscles postmortem and its relationship with meat quality. Asian-Australas J Anim Sci.

